# Ubiquitin carboxyl-terminal hydrolase isozyme L5 inhibits human glioma cell migration and invasion via downregulating SNRPF

**DOI:** 10.18632/oncotarget.23071

**Published:** 2017-12-07

**Authors:** Jiafeng Ge, Weiwei Hu, Hui Zhou, Juan Yu, Chongran Sun, Weilin Chen

**Affiliations:** ^1^ Institute of Immunology, Zhejiang University School of Medicine, Hangzhou 310058, China; ^2^ Department of Neurosurgery, Second Affiliated Hospital, Zhejiang University School of Medicine, Hangzhou 310058, China; ^3^ Department of Immunology, Shenzhen University School of Medicine, Shenzhen 518060, China

**Keywords:** UCHL5, glioma, SNRPF, migrationl, invasion

## Abstract

Ubiquitin C-terminal Hydrolase-L5 (UCH-L5/UCH37), a member of the deubiquitinases (DUBs), suppresses protein degeneration via removing ubiquitin from the distal subunit of the polyubiquitin chain. The activity of UCH-L5 is enhanced when UCH-L5 combines with proteasome 19S regulatory subunit by Rpn13/Admr1 receptor and inhibited when UCH-L5 interacts with NFRKB. But the role of UCH-L5 in gliomas remains unknown. In this study, analysis of 19 frozen and 51 paraffin-embedded clinic pathological cases showed that UCH-L5 expression in glioma tissues was lower than normal brain tissues. *In vitro*, we found that UCH-L5 could inhibit migration and invasion of U87MG and U251 cells. It has been reported that the expression of SNRPN, SNRPF, and CKLF was abnormal in gliomas or other tumors. We also found that SNRPF-siRNA, SNRPN-siRNA and CKLF-siRNA could inhibit migration and invasion of U87MG cells. And knockdown of UCH-L5 expression improved both mRNA expression and protein level of SNRPF. The relationship between UCH-L5 and SNRPF was further confirmed in 293T cells. Our study showed that UCH-L5 could inhibit migration and invasion of glioma cells via down regulating expression of SNRPF. And the above findings suggest that UCH-L5 may inhibit occurrence and metastasis of gliomas.

## INTRODUCTION

Gliomas are the most common primary brain tumors and are thought to arise from a neural stem cell [1]. About 30% of all brain tumors and 80% of all malignant brain tumors are gliomas [2]. Gliomas are great threats to human health for the high incidence rate, low cure rate and poor prognosis [3]. Gliomas are graded on the basis of the type of glial cells (astrocytes, oligodendrocytes or ependymal cells) from which they originate [4]. According to the malignant degree of cells, gliomas are classified as low-degree (WHO I-II degree) and high-degree (WHO III -IV degree) gliomas [5]. Treatments for gliomas are combination approaches, using surgery, radiation therapy and chemotherapy [6, 7]. However, mechanisms of occurrence, proliferation, migration, and invasion in glioma cells remain unknown. Therefore, it is important to study the molecular mechanisms in gliomas, and novel biomarkers should be screened for diagnosing of glioma patients.

Proteins are normally degraded by ubiquitin proteasome system (UPS) and autophagy [8]. The major protein disposal system is the UPS [9]. UPS exists in almost all eukaryotic cells, and consists of ubiquitins, ubiquitin like proteins, ubiquitin activating enzyme E1, ubiquitin conjugating enzyme E2 and ubiquitin ligase E3 and proteasome [8, 9]. Ubiquitin (Ub) is a highly conserved protein of 76 amino acids that is covalently linked to target proteins altering their localization, function, or stability [9, 10]. Deubiquitinases (DUBs) is a group of a large number of proteases, which can remove ubiquitins from the proteins that are modified with ubiquitins [11].

According to the homology and the functional mechanism of DUBs, we mainly divided them into 5 categories, namely Ub C-terminal hydrolase (UCH), Ub specific protease (USP), ovarian tumor protease (OTU), Josephin/Machado–Joseph disease protease (MJD) and JAB1/MPN/MOV34 metalloenzyme (JAMM) [11, 12]. UCH family is composed of four DUBs known as UCHL1, UCHL3, UCH-L5, and Bap1, they have close catalytic subunits that containing a cysteine-histidine-aspartic acid catalytic triad [12]. UCH-L5 is a 36KD protein containing 322 amino acids and is conserved from fungi to humans. UCH-L5 consists of two functional domains, a catalytic domain (UCH-domain) and a C-terminal domain (tail-domain) [13]. UCH-L5 has been regarded as part of the proteasome and INO80 complex. The activity of UCH-L5 is enhanced when UCH-L5 combines with proteasome 19S regulatory subunit by Rpn13/Admr1 receptor [14]. While the activity of UCH-L5 is inhibited when UCH-L5 interacts with NFRKB, a component of INO80 complex, by combination and deubiquitination [15]. The function of UCH-L5 in maintaining genome integrity via deubiquitinating NFRKB, protecting it from degradation has been reported [16].

A spliceosome is a kind of enzyme in eukaryotic cells. It removes introns from a transcribed pre-mRNA. The enzyme comprises of more than 100 associated proteins and 5 small nuclear ribonucleoproteins including U1, U2, U4, U5 and U6 [17]. Smith proteins (Sm proteins) are a family of RNA-binding proteins found virtually in every cellular organism. U1, U2, U4 and U5 are found to be tightly bound to Sm proteins. SNRPF, SNRPN, SNRPG, SNRPD1, SNRPD2, SNRPD3, SNRPB and SNRPE constitute a ring structure named Sm ring [18]. Sm proteins have also been reported to be involved in mRNA decapping and decay [19].

In the current study, we found that UCH-L5 was down expression in glioma tissue. Furthermore, we discovered that UCH-L5 could inhibit cell migration and invasion of glioma cell lines through downregulating SNRPF, a factor of Sm protein ring in the spliceosome. According to the abnormal protein level in high and low degree glioma [20], and mass spectra (MS) result after UCH-L5 overexpression [21], SNRPF, SNRPN and CKLF were as potential target genes of UCH-L5. We found that SNRPF, SNRPN and CKLF could inhibit migration and invasion of U87MG cells. Because SNRPF and SNRPN belonged to Sm gene family, we speculated that UCH-L5 might regulate mRNA level of Sm family. Then we examined mRNA level of other Sm genes including SNRPN, SNRPG, SNRPD1, SNRPD2, SNRPD3, SNRPB and SNRPE in U87MG cells. And we found UCH-L5 downregulated mRNA level of other Sm genes.

## RESULTS

### UCH-L5 is down expressed in glioma

**Figure 1:**
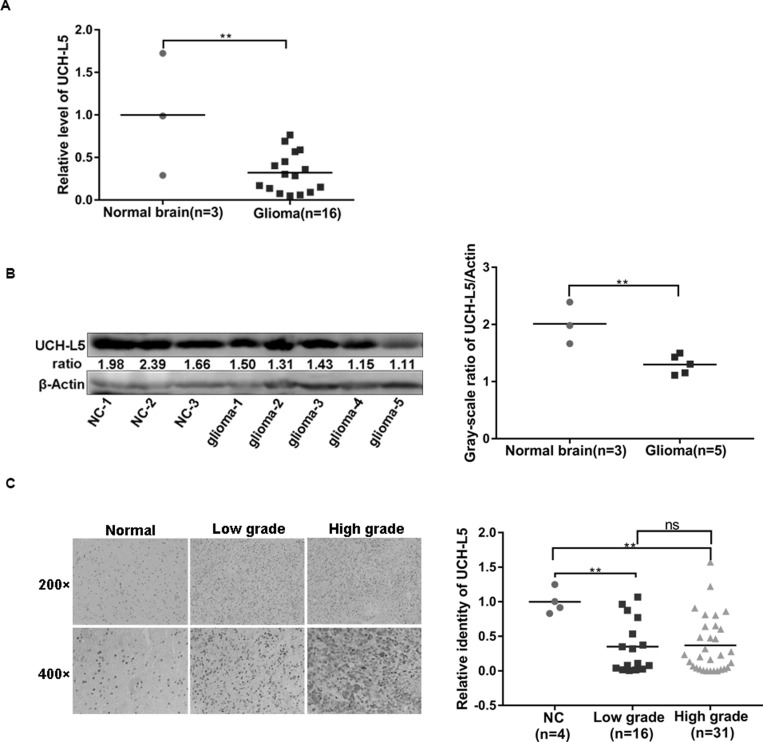
UCH-L5 is down expressed in gliomas (**A**) Relative mRNA level of UCH-L5 in normal brain tissues and glioma tissues examined by RT qPCR, ^**^P < 0.01. (**B**) Protein level of UCH-L5 in normal brain and glioma tissues detected by Western blot assays, and ratios of gray scale showing the difference in the picture,^**^P < 0.01. (**C**) Representative images of IHC analysis of low-grade and high-grade gliomas and normal brain tissues with UCH-L5 antibody, ^**^P < 0.01. Gliomas’ grades were classified as low-grade and high-grade gliomas according to WHO system (2007). Average optical density of UCH-L5 was calculated using imageJ software.

19 frozen samples including 3 normal brain tissues and 16 glioma tissues were analyzed for UCH-L5 expression by real-time quantity PCR (RT qPCR), and 3 normal brain tissues and 5 glioma tissues picked up randomly were carried out for UCH-L5 expression by Western blot. The results showed that mRNA level (Figure 1A) and protein level (Figure 1B) of UCH-L5 in glioma tissues were lower than normal brain tissues. And significant difference of UCH-L5 expression in normal brain tissues and glioma tissues was further approved by Immunohistochemistry (IHC) in Tissue microarray (TMA) (Figure 1C). These results indicated that low UCH-L5 expression was positively correlated with the occurrence of gliomas, but there was no difference between low-grade gliomas and high-grade gliomas (Figure 1C). The detailed information of frozen samples and paraffin-embedded samples were showed in Supplementary Table 1 and Supplementary Table 2 in Supplementary Material.

### Knockdown expression of UCH-L5 has no significant impact on apoptosis and cell cycle distribution in human glioma cells

**Figure 2:**
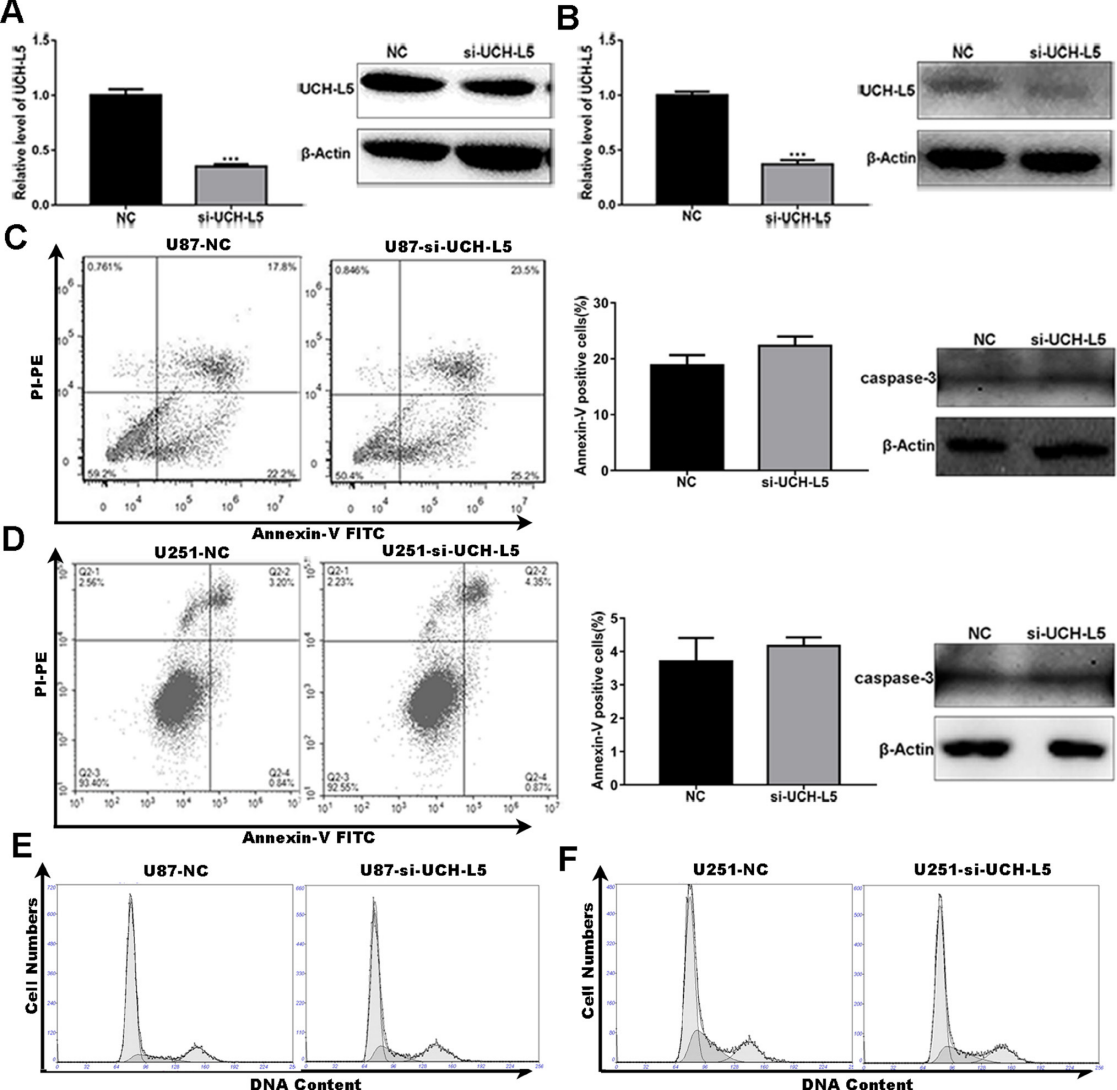
Knockdown of UCH-L5 expression has no effect on apoptosis and cell cycle distribution in human glioma cells (**A**) Analysis of UCH-L5 expression in U87MG cells treated with control scramble-siRNA or UCH-L5-siRNA determined by RT qPCR and Western blot. ^***^P < 0.001. (**B**) Analysis of UCH-L5 expression in U251 cells treated with control scramble-siRNA or UCH-L5-siRNA determined by RT qPCR and Western blot, ^***^P < 0.001. (**C**, **D**) U87MG cells (C) or U251 cells (D) were transfected with scramble-siRNA or UCH-L5-siRNA for 48 hours and followed double-stained with Annexin V and PI and analyzed by flow cytometry. The graphical representations of percentages of apoptotic cells were presented. And the protein levels of cleaved caspase-3 in U87 MG and U251 cells treated with or without UCH-L5-siRNA were analyzed by Western blot. (**E**, **F**) U87MG cells (E) or U251 cells (F) were transfected with scramble-siRNA or UCH-L5-siRNA for 48 hours and then stained with propidium iodide (PI). The DNA content was analyzed by flow cytometry. Percentages of cells in G0/G1, S, and G2/M phase were calculated using Multicycle software.

To further investigate the functions of UCH-L5 in gliomas, we firstly designed UCH-L5-siRNA (5′-GGAGACUGUAUGAAUUAGATT-3′), and it knocked down UCH-L5 efficiently in U87MG cells and U251 cells which were examined by RT qPCR and Western blot. Knockdown efficiency was about 70% in U87MG (Figure 2A) and 60% in U251 cells (Figure 2B). Flow cytometry showed that UCH-L5-siRNA had no significant impact on apoptosis of U87MG cells (Figure 2C) and U251 cells (Figure 2D). And there was also no difference between control group and group treated with UCH-L5-siRNA in apoptosis percentage and caspase-3 protein level. We also found that UCH-L5-siRNA had no significant impact on the cell cycle of U87MG cells (Figure 2E) and U251 cells (Figure 2F).

### Knockdown expression of UCH-L5 by siRNA promotes migration and invasion of human glioma cells

Since metastasis and recurrence represent the main malignant characteristics of high-grade glioma. We found that knockdown of UCH-L5 promoted the cell capability to migrate and invade in both U87MG and U251 cells.

**Figure 3:**
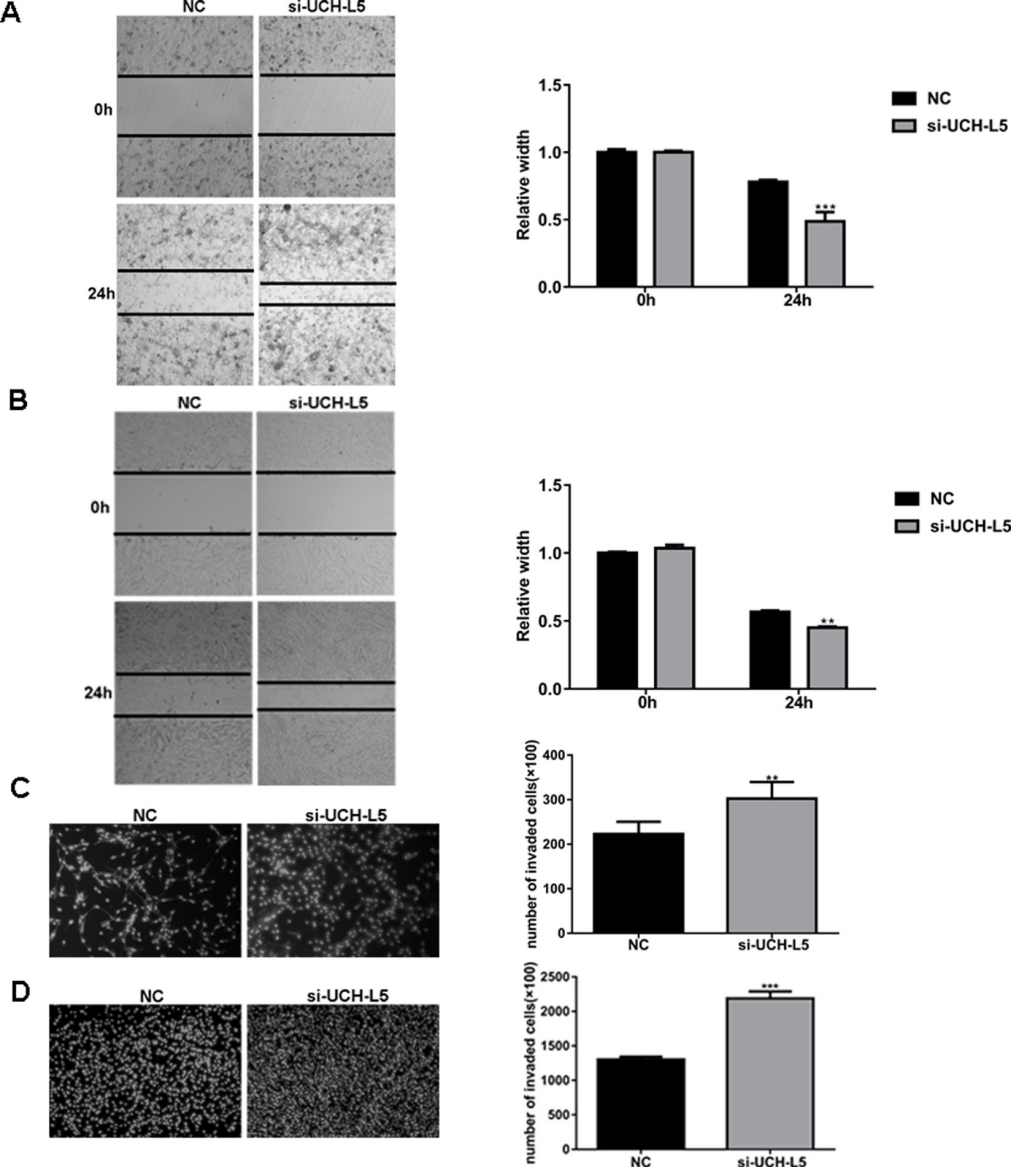
Knockdown of UCH-L5 expression by UCH-L5-siRNA promotes migration and invasion of glioma cells (**A**, **B**) Wound healing migration assay of U87MG (A) or U251 cells (B) in bright-field. Cells migrating into wound and bright-field images were captured at the indicated times after wounding by microscopy (100x). Bar graphs showing the relative width of gaps after scratching and data are represented as means ± SE from three independent experiments with significant differences from NC group in 24 h designated as ^**^P < 0.01, ^***^P < 0.001. (**C**, **D**) Transwell invasion assay of U87MG cells (C) or U251 cells (D), cells were seeded in DMEM without FBS in the upper compartment of transwell chambers which were added into 50 μl Matrigel firstly; lower chambers were filled with DMEM containing 20% FBS. The bottom sides of the filters were stained with DAPI to count the cells that migrated across the filter. Representative images are shown. Migrating cells were viewed under a microscope (100x), data are represented as means ± SE from three independent experiments with significant differences from control designated as ^**^P < 0.01, ^***^P < 0.001.

In a scratch-wound assay, scratch widths were measured every 12 h and width of the wound area of U87MG cells (Figure 3A) and U251 cells (Figure 3B) treated with UCH-L5-siRNA decreased markedly in 24 h,^***^P < 0.001, ^**^P < 0.01. In an invasion assay, the number of invading U87MG cells increased from 223 ± 19 cells per field for control to 316 ± 79 cells per field for cells treated with UCH-L5-siRNA, ^**^P < 0.01 (Figure 3C), and the numbers of invading U251 cells increased from 1303 ± 43 cells per field for control to 2173 ± 148 cells per field for cells treated with UCH-L5-siRNA, ^*^P < 0.05 (Figure 3D). These data indicated that reducing the expression of UCH-L5 improves the migratory and invasive abilities of glioma cell lines *in vitro*.

### Overexpression of UCH-L5 by Lentivirus infection inhibits migration and invasion of human glioma cells

We also found that overexpression of UCH-L5 by Lentivirus infection inhibits the cell capability to migrate and invade in both U87MG and U251 cells.

**Figure 4:**
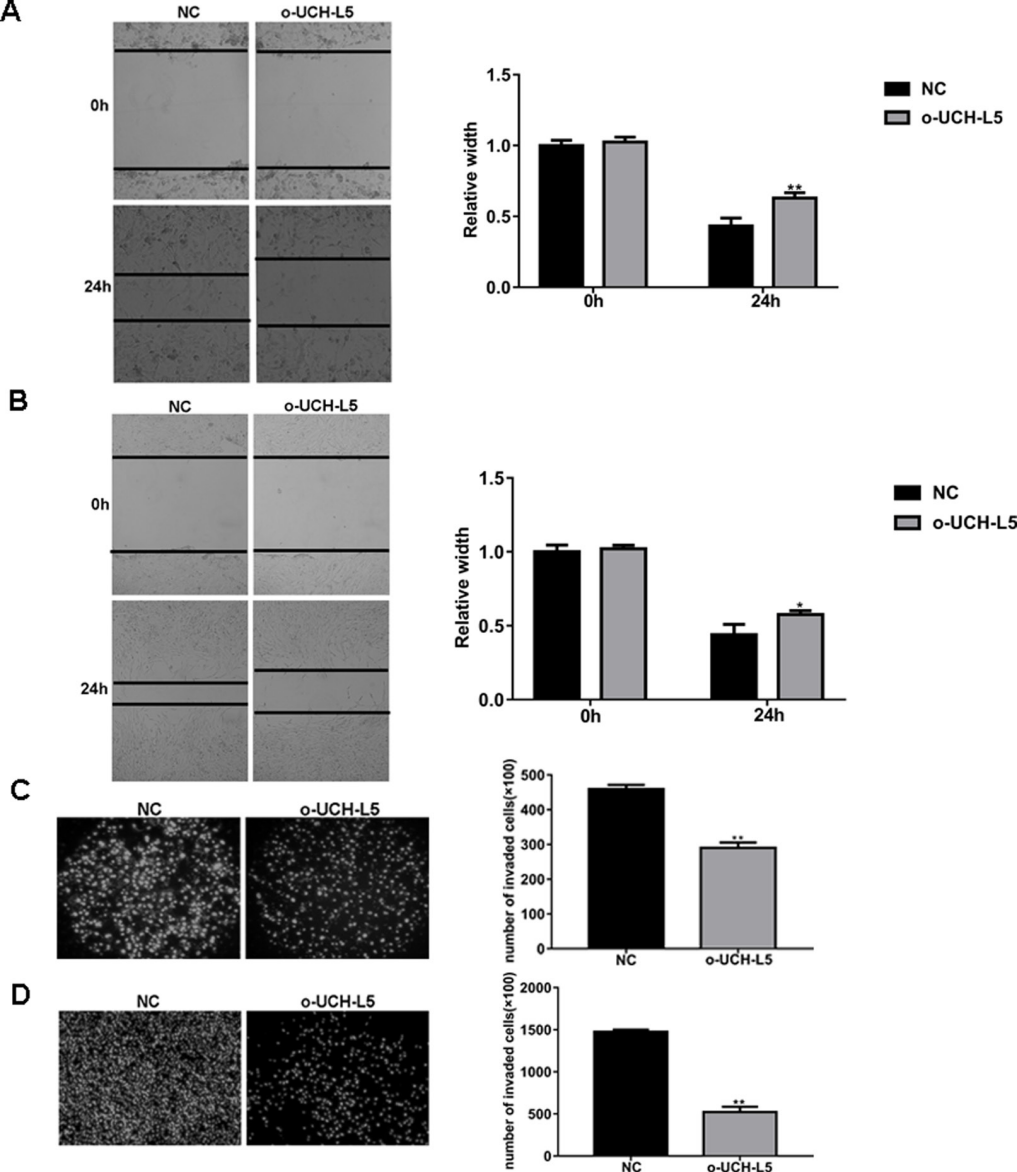
Overexpression of UCH-L5 by lentivirus infection inhibits migration and invasion of glioma cells (**A**, **B**) Wound healing migration assay of U87MG (A) or U251 cells (B) in bright-field. Cell migration into wound and bright-field images were captured at the indicated times after wounding by microscopy (100x). Bar graphs showing the relative width of gaps after scratching and data are represented as means ± SE from three independent experiments with significant differences from NC group in 24 h designated as ^*^P < 0.05, ^**^P < 0.01. (**C**, **D**)Transwell invasion assay of U87MG (C) or U251 cells (D), cells were seeded in DMEM without FBS in the upper compartment of transwell chambers which were added into 50 μl Matrigel firstly; lower chambers were filled with DMEM containing 15% FBS. The bottom sides of the filters were stained with DAPI to count the cells that migrated across the filter. Representative images are shown. Migrating cells were viewed under a microscope (100x), data are represented as means ± SE from three independent experiments with significant differences from control designated as ^**^P < 0.01.

In a scratch-wound assay, scratch widths were measured every 12 h, and width of the wound area of stable UCH-L5-overexpressing U87MG cells (Figure 4A) and stable UCH-L5-overexpressing U251 cells (Figure 4B) increased significantly in 24 h, ^**^P < 0.01, ^*^P < 0.05. In an invasion assay, the number of invading U87MG cells decreased from 411 ± 27 cells per field for control to 302 ± 22 cells per field for stable UCH-L5-overexpressing U87MG cells, ^**^P < 0.01 (Figure 4C), and the numbers of invading U251 cells decreased from 1476 ± 18 cells per field for control to 522 ± 45 cells per field for stable UCH-L5-overexpressing U251 cells, ^*^P < 0.05 (Figure 4D). These data indicated that overexpression of UCH-L5 inhibits the migratory and invasive abilities of glioma cell lines *in vitro*.

### Knockdown expression of SNRPN, SNRPF and CKLF inhibits migration and invasion of U87MG cells

**Figure 5:**
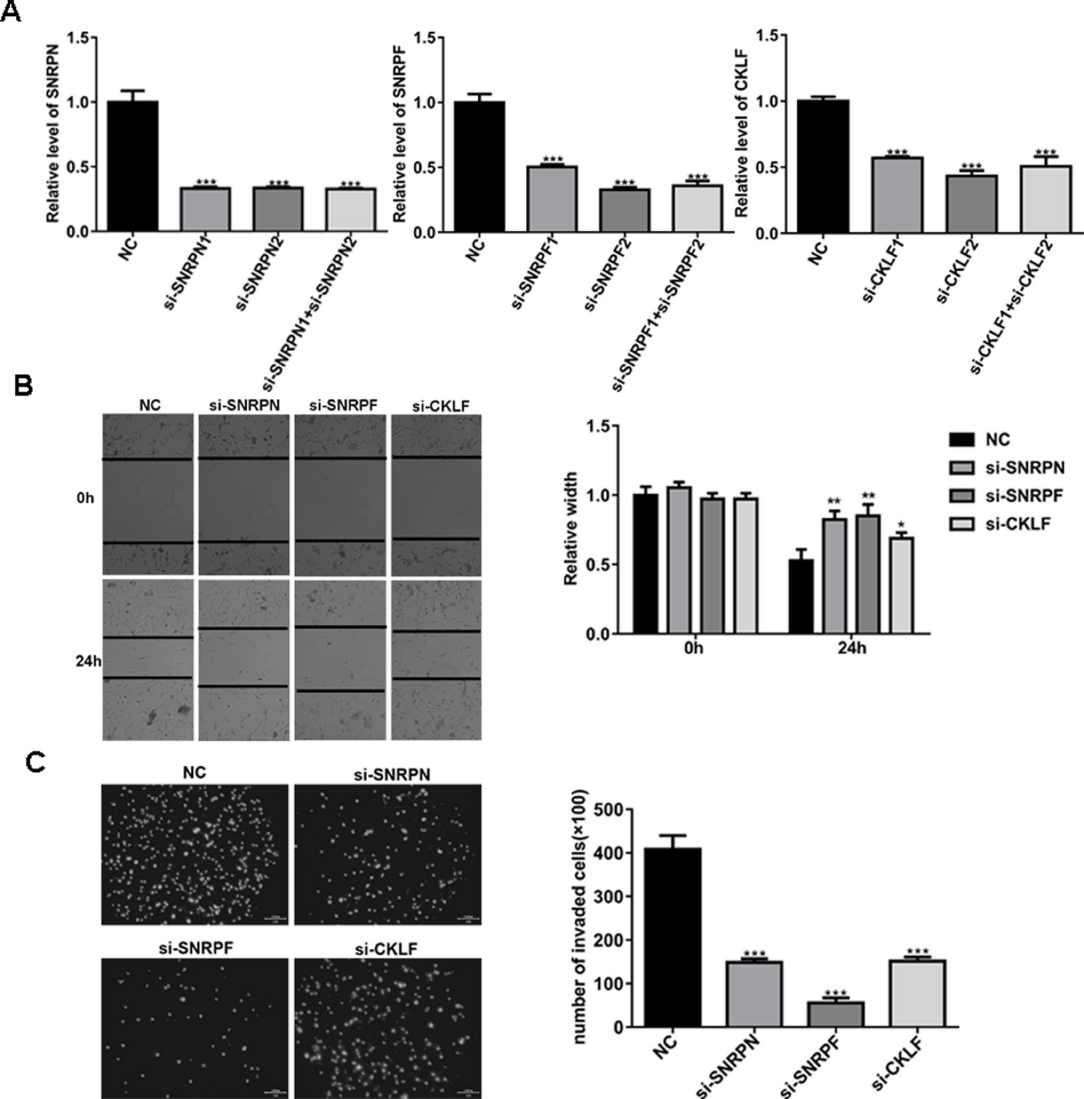
Knockdown of SNRPN, SNRPF or CKLF expression inhibits migration and invasion of U87MG cells (**A**) Analysis of knockdown efficiency of SNRPN, SNRPF, and CKLF in U87MG cells treated with SNRPN-siRNA, SNRPF-siRNA or CKLF-siRNA determined by RT qPCR. Compared with NC, ^***^P < 0.001. (**B**) Scratch-wound assay of U87MG cells treated with SNRPN-siRNA, SNRPF-siRNA and CKLF-siRNA. Wound widths were analyzed and compared with U87MG treated with scramble-siRNA using imageJ software, data are represented as means ± SE from three independent experiments with significant differences from NC group in 24 h designated as ^*^P < 0.05, ^**^P < 0.01. (**C**) Invasion assay of U87MG cells treated with SNRPN-siRNA, SNRPF-siRNA and CKLF-siRNA, invading cells were calculated and compared with U87MG cells treated with scramble-siRNA. Compared with NC, ^***^P < 0.001.

It has been reported that expression of SNRPN, SNRPF and CKLF is abnormal in glioma tissues. Therefore, we chose them as potential targeting genes of UCH-L5. Our results showed that SNRPF-siRNA, SNRPN-siRNA and CKLF-siRNA inhibited migration and invasion of U87MG cells. Knockdown efficiency of candidate genes in U87MG cells was examined by RT qPCR (Figure 5A).

In a scratch-wound assay, scratch widths were measured every 12 h. Comparing with U87MG cells treated with scremble-siRNA, width of the wound area of U87MG cells treated with SNRPF-siRNA, SNRPN-siRNA and CKLF-siRNA increased significantly in 24 h (Figure 5B). In an invasion assay, comparing with U87MG cells treated with scramble-siRNA, the number of invading U87MG cells treated with SNRPF-siRNA, SNRPN-siRNA and CKLF-siRNA decreased from 408 ± 32 per field for control to 149 ± 8, 57 ± 11 and 151 ± 10 cells per field for cells treated with SNRPF-siRNA, SNRPN-siRNA and CKLF-siRNA respectively (Figure 5C). These data indicated that knockdown of SNRPF, SNRPN and CKLF inhibits migration and invasion of U87MG cell *in vitro*.

### UCH-L5 regulates SNRPF expression

**Figure 6:**
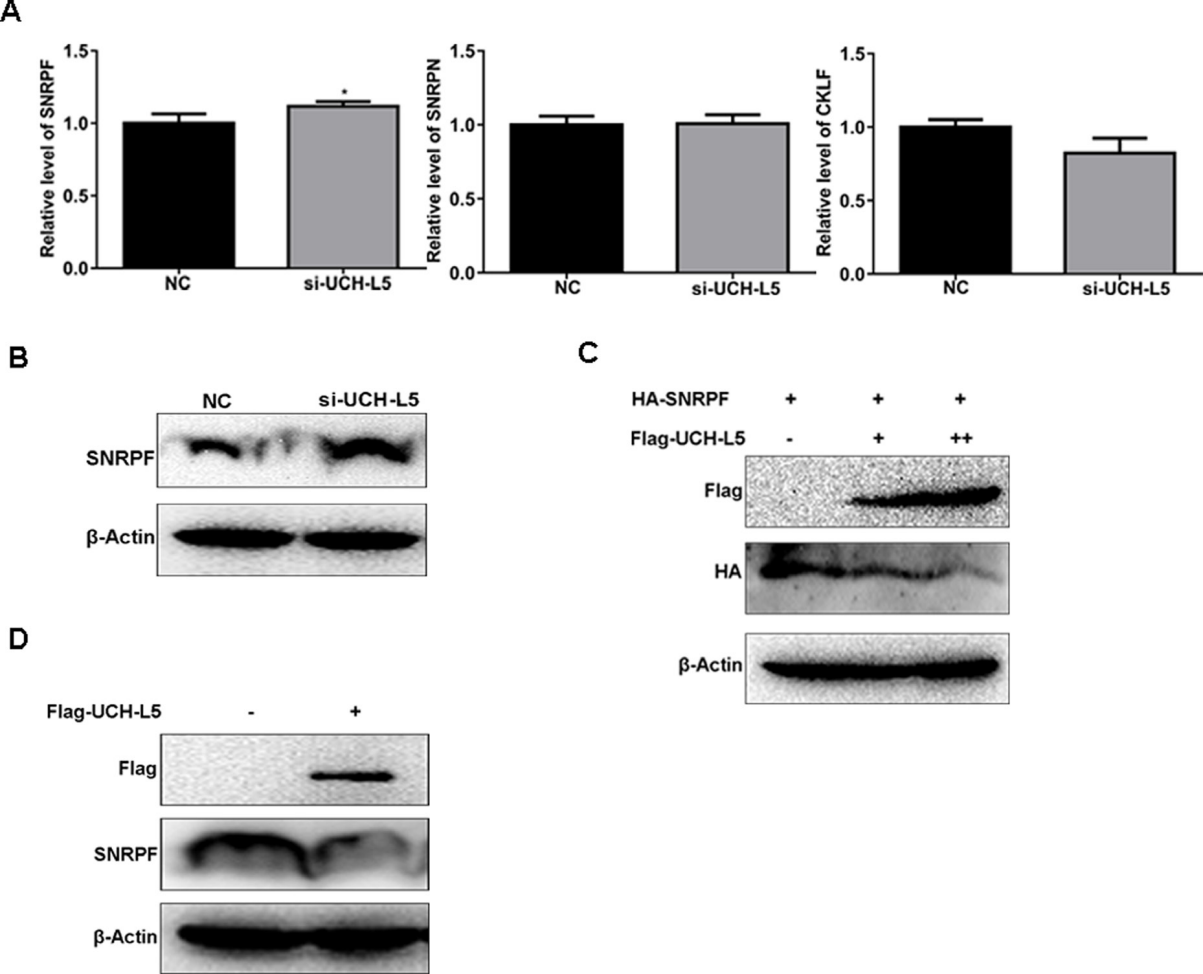
UCH-L5 downregulates SNRPF expression (**A**) Analysis of mRNA level of SNRPF, SNRPN and CKLF in U87MG cells treated with UCH-L5-siRNA determined by RT qPCR, showing only SNRPF mRNA expression was upregulated, compared with U87MG cells treated with scramble-siRNA, ^*^P < 0.05. (**B**) Analysis of the protein level of SNRPF in U87MG cells treated with UCH-L5-siRNA examined by Western blot. (**C**) Analysis of the protein level of HA-SNRPF after cotransfecting Flag-UCH-L5 and HA-SNRPF plasmids into 293T cells detected by Western blot. (**D**) Analysis of endogenous protein level of SNRPF after transfecting Flag-UCH-L5 in 293T cells.

To find the targeting gene of UCH-L5, firstly, we found that mRNA level of SNRPF was higher in U87MG cells treated with UCH-L5-siRNA than the control treated with scramble-siRNA, but mRNA level of SNRPN or CKLF do not change significantly (Figure 6A), ^*^P < 0.05. And the protein level of SNRPF also rose in U87MG cells treated with SNRPF-siRNA (Figure 6B). Then we cloned two plasmids including Flag-UCH-L5 and HA-SNRPF expressing plasmids respectively. We co-transfected Flag-UCH-L5 and HA-SNRPF plasmids into 293T cells and found HA-SNRPF expression decreased following the increasing of Flag-UCH-L5 expression (Figure 6C). And after transfecting Flag-UCH-L5, we also found endogenous protein level of SNRPF decreased in 293T cells (Figure 6D).

### SNRPF mRNA and protein expression are regulated by UCH-L5 in U87MG and U251 cells with stable UCH-L5 silencing and stable UCH-L5 overexpressing by lentivirus

**Figure 7:**
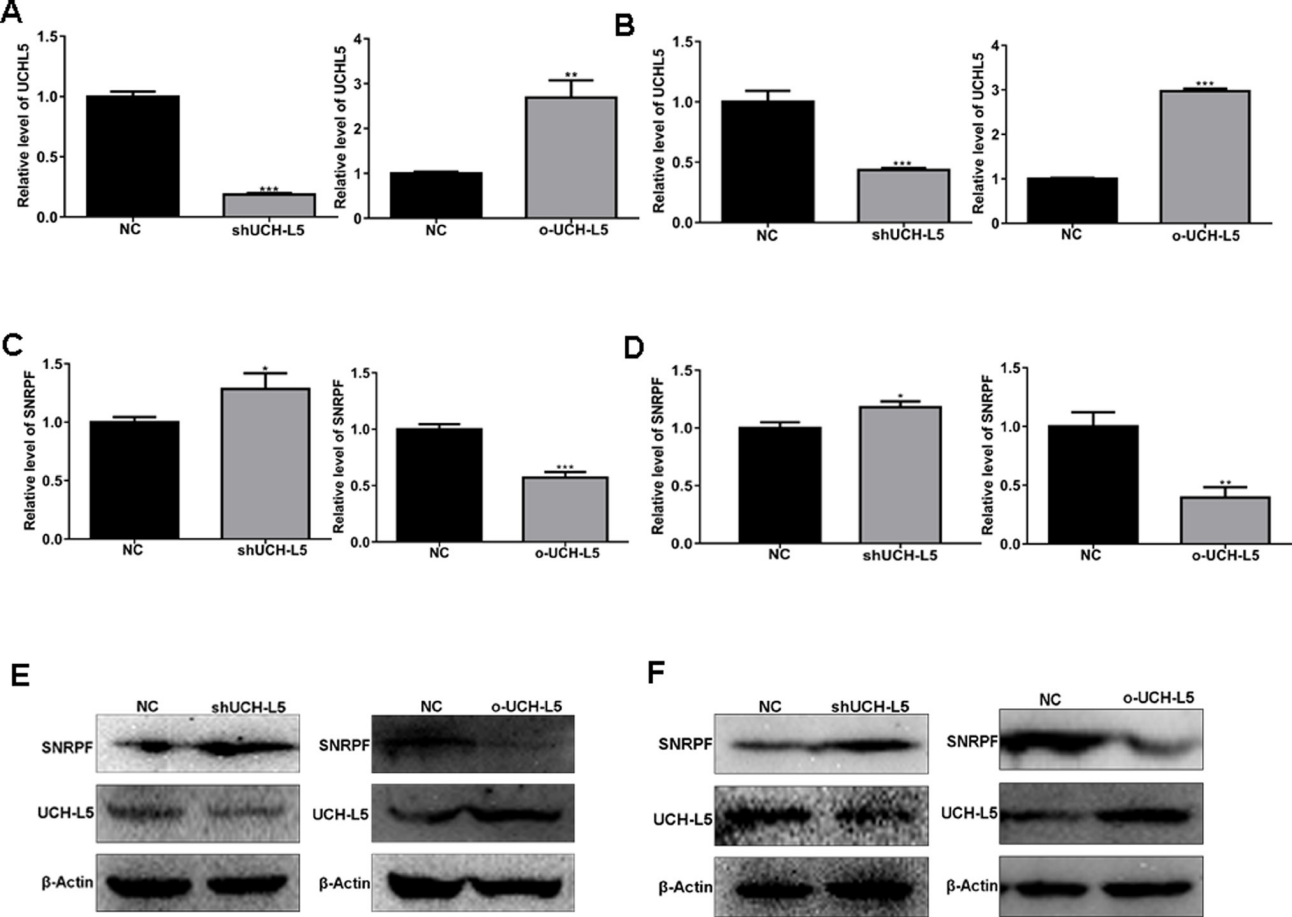
UCH-L5 downregulates mRNA and protein level of SNRPF in U87MG cells with stable UCH-L5 knockdown and stable UCH-L5 overexpressing by lentivirus (**A**) Analysis of UCH-L5 knockdown and overexpression efficiency by lentivirus infection in U87MG cells determined by RT qPCR. Compared with NC, ^**^P < 0.01, ^***^P < 0.001. (**B**) Analysis of UCH-L5 knockdown and overexpression efficiency by lentivirus infection in U251 cells determined by RT qPCR. Compared with NC, ^***^P < 0.001. (**C**) UCH-L5 downregulates mRNA level of SNRPF in U87MG cells determined by RT qPCR. Compared with NC, ^*^P < 0.05, ^***^P < 0.001. (**D**) UCH-L5 regulates mRNA level of SNRPF in U251 cells by RT qPCR detecting. ^*^P < 0.05, ^***^P < 0.001. (**E**) UCH-L5 downregulates protein level of SNRPF in U87MG cells confirmed by Western blot. (**F**) UCH-L5 downregulates protein level of SNRPF in U251 cells confirmed by Western blot.

To further confirm that UCH-L5 inhibits SNRPF expression, U87MG and U251 cells were subjected to analysis for lentivirus-mediated gene knockdown and overexpression. U87MG and U251 cells with infected efficiency of more than 90% were presented by GFP-expression, and stable knockdown and overexpression UCH-L5 of colonies were screened by puromycin as subsequent experimental objects. Lentivirus-mediated knockdown and overexpression efficiency were about 70% and 2 times in U87MG cells separately (Figure 7A). And the lentivirus-mediated knockdown and overexpression efficiency were 60% and 3 times in U251 cells separately (Figure 7B). Accordingly, we found UCH-L5 inhibited mRNA expression (Figure 7C and 7D) and protein level (Figure 7E and 7F) of SNRPF both in U87MG cells and U251 cells significantly.

Because SNRPF, SNRPN are members of Sm family, we detected mRNA level of other Sm genes in U87MG cells. In stable UCH-L5 knockdown and stable UCH-L5 overexpressing U87MG cells, we found that knockdown UCH-L5 expression upregulated mRNA level of Sm genes except for SNRPN (Supplementary Figure 1A), while UCH-L5 overexpression downregulates mRNA level of Sm genes in U87MG cells (Supplementary Figure 1B). Thus, we thought UCH-L5 may inhibit transcription of Sm genes.

## DISCUSSION

Gliomas are the most common primary tumors of the central nervous system (CNS) [1]. Gliomas have become one of the serious diseases that endanger human health with gradually increasing incidence [3]. Gliomas are generally classified as low-grade and high-grade gliomas according to the World Health Organization (WHO) system (2007) that is largely based on pathological features [5]. Low-grade gliomas including Grade I and II tumors are considered non-malignant, and high-grade glioma including Grade III and IV tumors are malignant. And the subsets are phenotyped and genotyped to define [4, 5]. Patients with high-degree gliomas especially glioblastoma (GBM) have poor diagnose and survival [6, 7]. Current treatments, including radiation and chemotherapy with temozolomide, provide a better survival benefit [22]. Therefore, it is of great significance to explore the pathogenesis of gliomas. And further understanding of glioma biology and treatment is highly needed.

The common roles of DUBs in various cellular processes are now well understood and new functions are continued to emerge gradually. DUBs are involved in degradative and non-degradative signaling such as signal pathway activation [23], gene transcription [24], DNA repair and replication [16], and new findings in inflammation and autoimmunity [25, 26]. Thus, targeting DUBs could ascertain influences on the fate of proteins. DUBs are also associated with cancers [27, 28] and other diseases [29, 30]. Nowadays, more and more studies are focused on targeting the activity of deubiquitinases especially inhibition of proteasome deubiquitinating activity as a novel cancer therapy [31, 32].

Ubiquitination is regulated by DUBs. At present, there are lots of researches who proved that ubiquitination is involved in the regulation of glioma [33, 34]. And it has been reported that UCH family plays a potential role in oncogenesis [35, 36]. We investigated that UCH-L5 might directly be implicated in the regulation of glioma development, which will be helpful to find a potential therapeutic target for gliomas. UCH-L5 is regarded as components of two complexes, proteasome and INO80 complex. These two companions share a conserved Deubiquitinase Adaptor (DEUBAD) domain that binds to a unique C-terminal region in UCH-L5, termed as the UCH-L5-like domain (ULD) [14]. UCH-L5 slowly shortens ubiquitin chains, allowing the attached protein to be released from the proteasome if there is a delay in efficient degradation. UCH-L5 also regulates DNA double-strand breaks (DSBs) resection and repair by homologous recombination through protecting its interactor, NFRKB, from degradation [16]. Therefore, UCH-L5 prompts the maintenance of genome integrity might be potential as a therapeutic target for cancers. The essential cysteine protease UCH-L5 is activated by proteasome ubiquitin receptor RPN13 (ADRM1) or inhibited by chromatin remodeling complex component INO80 (NFRKB) [37]. Sahtoe *et al.* and Vander Linden *et al.* uncover the detailed mechanism of deubiquitination domains in RPN13 (ADRM1) and INO80G (NFRKB), can either activate or inhibit UCH-L5 [15, 37].

UCH-L5 is also linked to TGF-β signaling [38], and overexpresses in several carcinomas [39–41]. Fang Y *et al*. found UCH-L5 promotes cell migration and invasion via interacting and deubiquitinating splicing factor PRP19 in hepatocellular carcinoma [21]. Therefore, UCH-L5 might play a potential role in oncogenesis.

However, in our study, we found that the expression of UCH-L5 both in low-grade gliomas and high-grade gliomas were lower than normal brain tissues. Furthermore, we found that knockdown of UCH-L5 expression promotes the migration and invasion of U87MG and U251 cells. And overexpression of UCH-L5 inhibits the migration and invasion of U87MG and U251 cells. These findings are different from the previous studies in other cancers. Therefore, we conclude that UCH-L5 plays a negative regulatory role in gliomas. However, further studies showed that the downregulation of UCH-L5 has no effect on the apoptosis and cell cycle of U87MG and U251 cells. These results suggest that UCH-L5 may inhibit the migration and invasion of glioma cells to inhibit the occurrence of glioma.

A spliceosome is a large and complex molecular machine found primarily within the splicing speckles of the cell nucleus of eukaryotic cells. It consists of about 100 proteins and 5 Small nuclear ribonucleic acid (snRNAs), including U1, U2, U4, U5, and U6 [42]. The spliceosome removes introns from a transcribed pre-mRNA, a type of primary transcript [43]. Spliceosome assembly processing includes four steps, from the early complexes (E complex) to precursor spliceosome (A complex) to mature spliceosome (B complex and C complex) [44]. It has been reported that the disruption of components and the formation steps of spliceosome will increase the occurrence of cancers and other diseases [45]. Until now, the structure of spliceosome in every formation step is more and more clear, and the functions of components will be further studied.

We screened some candidate genes including SNRPF, SNRPN, and CKLF from previous study [20, 21]. SNRPF and SNRPN are two of members of Sm proteins ring as part of a spliceosome. Sm ring consists of a set of uridine-rich small nuclear proteins including SNRPE, SNRPG, SNRPD3, SNRPB, SNRPN, SNRPD1, SNRPD2, and SNRPF. They arrange in decreasing order of size and bind to small nuclear RNA (snRNA) as a part of U1, U2, U4, U5 and U6 [46]. SNRPF has been shown to interact with DDX20 [47], SNRPD2 and SNRPE [48]. It has been only found that casepase-8 and other caspase family members implicate the cleavage of the SNRPF protein during apoptosis [49]. In our study, the scratch and invasion assay results showed that SNRPF, SNRPN, and CKLF could inhibit the migration and invasion of U87MG cells, and knockdown of UCH-L5 expression only unregulated the gene level of SNRPF but not mRNA expression of SNRPN or CKLF in U87MG cells. We also found that knockdown of UCH-L5 could upregulate the mRNA and protein level of SNRPF, while overexpression of UCH-L5 downregulated the mRNA and protein level of SNRPF in U87MG cells infected by Lentivirus. So UCH-L5 could inhibit glioma cell migration and invasion via downregulating SNRPF. Considering the function of UCH-L5 regulates DNA transcription and mRNA expression of the spliceosome components, the possible reason is that knockdown of UCH-L5 expression upregulates mRNA level of SNRPF which promots the splicing of downstream oncogenes, causing a promotion of oncogenic genes and tumorigenesis.

In previous study, the INO80 chromatin-remodeling complex has been implicated in DNA replication. And it has been reported that INO80 regulates gene transcription through binding to replication forks and promoting fork progression in human cells [24]. And BRCA1-associated protein-1 (BAP1), a homogeneous protein of UCH-L5, having a common ancestry, regulates normal DNA replication via stabilizing and recruiting INO80 to replication forks [24]. And UCH-L5 also interacts with NFRKB in INO80 complex, but it remains unknown that whether UCH-L5 interacts with other components of INO80 complex or not. Thus, the possible mechanism of UCH-L5 downregulates SNRPF expression may through interacting with NFRKB or other components of INO80 complex.

## MATERIALS AND METHODS

### Clinical specimens

A total of 19 frozen samples including 3 normal brain tissues and 16 glioma tissues, and 51 paraffin-embedded samples including 4 normal brain tissues, 16 low-grade gliomas and 31 high-grade gliomas were from the department of Neurosurgery of the Second Affiliated Hospital, Zhejiang University, School of Medicine (Hangzhou, China). The patients were diagnosed as gliomas by preoperative CT and MRI examinations, and tumor tissues were obtained by surgical removal. 3 normal brain tissues were obtained from patients with cerebral trauma and cerebral hemorrhage. Therefore, 19 frozen samples were subjected to the analysis of the different expressions of UCH-L5 between gliomas and normal brain tissues by RT qPCR and Western blot. And 51 paraffin-embedded tissues samples were subjected to the analysis of the different expressions of UCH-L5 by Immunohistochemistry. We classified gliomas as different types according to the morphology or origin of tumor cells, and subtypes were categorized according to the malignancy of cells. And we defined the Low-grade glioma and High-grade glioma according to the WHO classification of tumors of the central nervous system (2007). More information about frozen and paraffin-embedded samples was showed in Table 1 and Table 2 in supplementary data2. The study was approved by the ethics committee of the Zhejiang University (Hangzhou, China).

### Immunohistochemical staining of TMA

Paraffin-embedded tumor tissues were cut into 5μm paraffin sections, Sections for Immunohistochemical SABC staining were dewaxed in xylene, soaked with 100%, 95%, 90%, 80%, 70% ethanol and H_2_O. Endogenous catalase activity was inactivated by H_2_O_2_. Sections were set in citrate buffer (PH6.0) and heated to fixed antigen. Then Sections were put in a wet box and blocked by 5%BSA, and incubated with 1:100 dilution of rabbit anti-UCH-L5 antibody (Santa Cruz, USA) at 4°C overnight, and with the biotinylated goat anti-rabbit antibody with the streptavidin-peroxidase conjugate. Finally, 3′, 3′-diaminobenzene (DAB) was used for color reaction. All staining results were observed and evaluated under a light microscope. The percentage of positively stained cells and the staining intensity were calculated and analyzed by using imageJ software.

### Cell culture

U87MG, U251, and 293T cells (Cell Bank of the Chinese Academy of Sciences, Shanghai, China) were cultured in DMEM with 10% fetal bovine serum (FBS) and 1% myocilin.

### RNA interference

10^5^ U87MG or U251 cells were inoculated on the 12-well or 6-well cell culture plate and placed in 37°C, 5% CO_2_ incubator overnight. After the screening concentration of siRNA, 30 nM UCH-L5-siRNA for U87MG and U251 cells, and 30 nM SNRPF-siRNA, SNRPN-siRNA, or CKLF-siRNA for U87MG cells were added to 200 μl DMEM medium, adding 4 μl (12 well plate)or 6 μl (6 well plate) interfering reagent and reacting at room temperature (RT) for 15 minutes, then added to transfection complexes into plates and following experiments were continued after 48 hours. The siRNA sequences of candidate target genes including: SNRPN-1:GCCAAAGAAUGCGAAGCAATT, SNRPN-2:UCUUCAUUGGCACCUUUAATT;SNRPF-1:CCUUUCCUCAAUGGACUAATT, SNRPF-2:UCCCAAACCUUUCCUCAAUTT; CKLF-1: GCACU AACUGUGACAUCUATT, CKLF-2: GGCCUUUGCUUGAUAUUAUTT The RT qPCR primers include: for SNRPN: CCC AGC TTG CAT TGT TTC TAG (Forward), CAT CTT GCT ACT CTT GCC AAC (Reverse). for CKLF: TGC TCA TCG TAT CTG TGT TGG (Forward), AGC TTC CGG TAA ATA AGG GC (Reverse); for SNRPF: AGA GTA GCC TGC AAC ATT CG (Forward), GAT AGC CCT TGT ACT CCA TTC C (Reverse). And for ACTB: ACCTTCTACAATGAGCTGCG (Forward), CCT GGA TAG CAA CGT ACA TGG (Reverse).

### Plasmid, shRNA, and transfection

4shRNA(shRNA1:GTCCCGACTTGACACGATATTTTCAAGAGAAATATCGTGTCAAGTCGGGATTTTTT.shRNA2:GAGCCAGTTCATGGGTTAATTTTCAAGAGAAATTAACCCATGAACTGGCTTTTTTT.shRNA3:GGAGACTGTATCAATTAGATTTCAAGAGAATCTAATTCATACAGTCTCCTTTTTT.shRNA4:GTGAAGGTGAAATTCGATTTAATTCAAGAGATTAAATCGAATTTCACCTTCATTTTT) targeting UCH-L5 (NM001199261) were designed, and shRNA3 was indicated effective in U251 and U87MG cells. shRNA were inserted into lentivirus vector pLent-4 in 1 shRNA-GFP-Puro (Vigene. Inc. Shandong, China). Lentivirus mediated overexpression vector of UCH-L5 was subcloned from pDEST-LTR-N-FLAG-HA-UCH-L5-puro (Addgene, USA). The UCH-L5 ORF was inserted into pLent-EF1a-FH-CMV-GFP (Vigene. Inc. Shandong, China). These constructs were confirmed by DNA sequencing. Lentivirus was packaged in 293T cells and lentivirus titer was 1 × 10^8^/ml. 3 days after infection, U87MG and U251 cells with stable knockdown UCH-L5 and stable overexpressing UCH-L5 cell lines were screened by GFP and puromycin. The knockdown and overexpression efficiency of UCH-L5 were determined by RT qPCR and Western blot. pCMV-Tag 2B and pCMV-HA plasmid were purchased from Invitrogen (USA). UCH-L5 ORF was inserted into pCMV-Tag 2B, SNRPF ORF (NM_003095.2) was reversed from total RNA from U87MG cells, subcloned and inserted into pCMV-HA. ORF PCR primers sequences for UCH-L5 and SNRPF were:

for UCH-L5, 5′-CGCGGATCCATGACGGGCAATGCCGGG-3′ (forward) and 5′-CCGGAATTCTTTGGTTTCCTGAGCTTTCTTTGC-3′ (reverse); for SNRPF, 5′-CGGAATTCGGATGAGTTTACCCCTCAATCCCAAAC-3′ (forward) and 5′-GGGGTACCTTCTCTCATTTCCCCATCTTCTTC-3′ (reverse).

### Western blot

Cells were lysed in cell lysis buffer (cell signaling technology, USA) with a protease inhibitor cocktail (BBI Life Sciences, China). Proteins were separated by 10% or 12% SDS-PAGE and transferred onto PVDF membranes. After blocking in 5% non-fat milk, the membranes were incubated with primary antibodies overnight at 4°C and detected by incubating with specific secondary antibodies (Jackson ImmunoResearch, USA) for 2 h at room temperature. Then protein bands were exposed with ECL chromogenic substrate. β-Actin (Huaan biotech, China) served as an internal loading control. Other primary anti-human antibodies include UCH-L5 (Santa Cruz, USA), SNRPF (abcam, USA), Flag-tag (MultiSciences Biotech, China) and HA-tag (Abmart, China).

### Cell cycle assay

2 × 10^5^ U87MG or U251 cells were incubated into 6-well plates, adherent cells were treated with serum free medium for 24 hours to keep cells in the same period, continuing to cultivate 48 hours after interference treatment. Cells were digested with trypsin and centrifuged in 300 g for 5 minutes. After suspending in PBS buffer and centrifuging in 300 g for 5 min, cells were suspended in 500 μL PBS buffer and fixed with 4.5 ml 70% cold ethanol at 4°C for 12 h. Then centrifuging in 800 g for 5 minutes at 4°C and suspending with 1 ml PBS buffer containing 10% serum. After centrifuging in 800 g for 5 minutes at 4°C again and adding 500 μl staining buffer (50 ug/ml PI, 100 μg/ml RNase A and 0.2%Triton X-100), cells were incubated at 37°C for 1 h in the dark. Flow analysis was carried out after adding 500 μl PBS buffer. Each sample had 3 repetitions accordingly.

### Cell apoptosis assay

U87MG and U251 cells were treated with scramble-siRNA or UCH-L5-siRNA, SNRPF-siRNA, SNRPN-siRNA, and CKLF-siRNA for 48 h, 1 × 10^5^ cells were trypsinized, centrifuged, washed with ice-cold PBS buffer. Absorbing the supernatant and resuspending cells with 100 μl Annexin V-FITC binding buffer. Then cells were incubated in 5 μl propidium iodide (PI) staining (PI dye and RNase A) solution and 5 μl Annexin V-FITC for 15 min at RT in the dark. Cell apoptosis was finally analyzed by flow cytometry within 1 h. Each sample had 3 repetitions.

### Cell migration assay

10^5^ U87MG or U251cells were seeded on the 12-well plate, U87MG and U251 cells were treated with scramble-siRNA, UCH-L5-siRNA, SNRPF-siRNA, SNRPN-siRNA, and CKLF-siRNA for 48 h. The cell monolayers were scraped with a sterile yellow micropipette tip to create a denuded area of constant width. Cells were washed with PBS buffer to remove cell debris, and cultured in DMEM with 2% FBS. The wound widths were photographed every 12 hours in the same area.

### Cell invasion assay

Cells were seeded on the 6-well plate, U87MG and U251 cells were treated UCH-L5-siRNA, SNRPF-siRNA, SNRPN-siRNA, and CKLF-siRNA for 48 h. Cells were digested, centrifuged and diluted to 5 × 10^5^/ml with serum free medium, and 100 μl cell suspensions were added to the top chamber with a matrigel-coat membrane. And DMEM containing 15% FBS was used as a chemoattractant in the lower chambers. After incubation for 48 h, cells were fixed with methanol and stained with 4’, 6-diamidino-2-phenylindole (DAPI). Then cells were counted in 5 independent fields with a light microscope.

### Statistical analysis

The identity of UCH-L5 in Immunohistochemical samples was tested using ImageJ software. Statistical significance was determined by *t*-test using Prism GraphPad software. *P* < 0.05 was considered statistically significant.

## SUPPLEMENTARY MATERIALS FIGURE AND TABLES



## Abbreviations

UCH-L5: Ubiquitin C-terminal Hydrolase-L5
DUBs: deubiquitinases
PBS: phosphate buffer saline
Ub: Ubiquitin
UPS: ubiquitin proteasome system
USP: Ub specific protease
Sm proteins: Smith protein
TMA: Tissue microarray
RT qPCR: real-time quantity PCR
PI: propidium iodide
DMEM: Dulbecco’s modified eagle medium
DAPI: 4′, 6-diamidino-2-phenylindole
snRNAs: Small nuclear ribonucleic acid
SNRPN: Small nuclear ribonucleoprotein polypeptide N
SNRPF: Small nuclear ribonucleoprotein polypeptide F
CKLF: chemokine like factor
MS: mass spectra
