# Supplementary material for: Ubiquitin carboxyl-terminal hydrolase isozyme L5 inhibits human glioma cell migration and invasion via downregulating SNRPF
Source: Oncotarget. 2017 Dec 7;8(69):113635–49. doi: 10.18632/oncotarget.23071 (PMC5768352; doi:10.18632/oncotarget.23071)
Supplement: Supplementary file 1 [file oncotarget-08-113635-s001.pdf]

Ubiquitin carboxyl-terminal hydrolase isozyme L5 inhibits human glioma cell migration and invasion via downregulating SNRPF

SUPPLEMENTARY MATERIALS

Supplementary Table 1: The characteristics of the frozen samples for RT qPCR and Western blot analysis

Variables	Normal tissues (N = 3)[*]	Glioma (N = 16)
Sex	0	7
Female	3	9
Male		
Age, year		
<18	0	1
≥18	3	15
Tumor classification	0	4[**]
Low-grade (I–II degree)		
High-grade (III–IV degree)	0	12[***]

[*]3 normal brain tissues come from the patients with cerebral hemorrhage and brain trauma.
[**]4 low grade gliomas include 1ganglioglioma, 2 diffuse astrocytoma, and 1 pilocytic astrocytoma
[***]12 high grade gliomas include 11glioblastoma, and 1anaplastic oligodendroglioma.

Supplementary Table 2: The characteristics of the paraffin-embedded samples for IHC analysis

Variables	Normal tissues (N = 4)[*]	Glioma (N = 47)
Sex		
Female	4	23
Male	0	24
Age, year		
<18	0	1
≥18	4	46
Tumor classification		
Low-grade (I–II degree)	0	16[**]
High-grade (III–IV degree)	0	31[***]

[*]4 normal brain tissues come from the patients with cerebral hemorrhage and brain trauma.
[**]16 low grade gliomas include 7 diffuse astrocytoma, 4 oligoastrocytomas, 3 ependymocytoma, 1 pilocytic astrocytoma, and 1 pleomorphic xanthoma astrocytoma.
[***]31 high grade gliomas include 21 glioblastoma, 6 anaplastic oligodendroglioma, 3 anaplastic astrocytoma, 1 anaplasticgangIioglioma, and 1 anaplastic ganglioglioma.

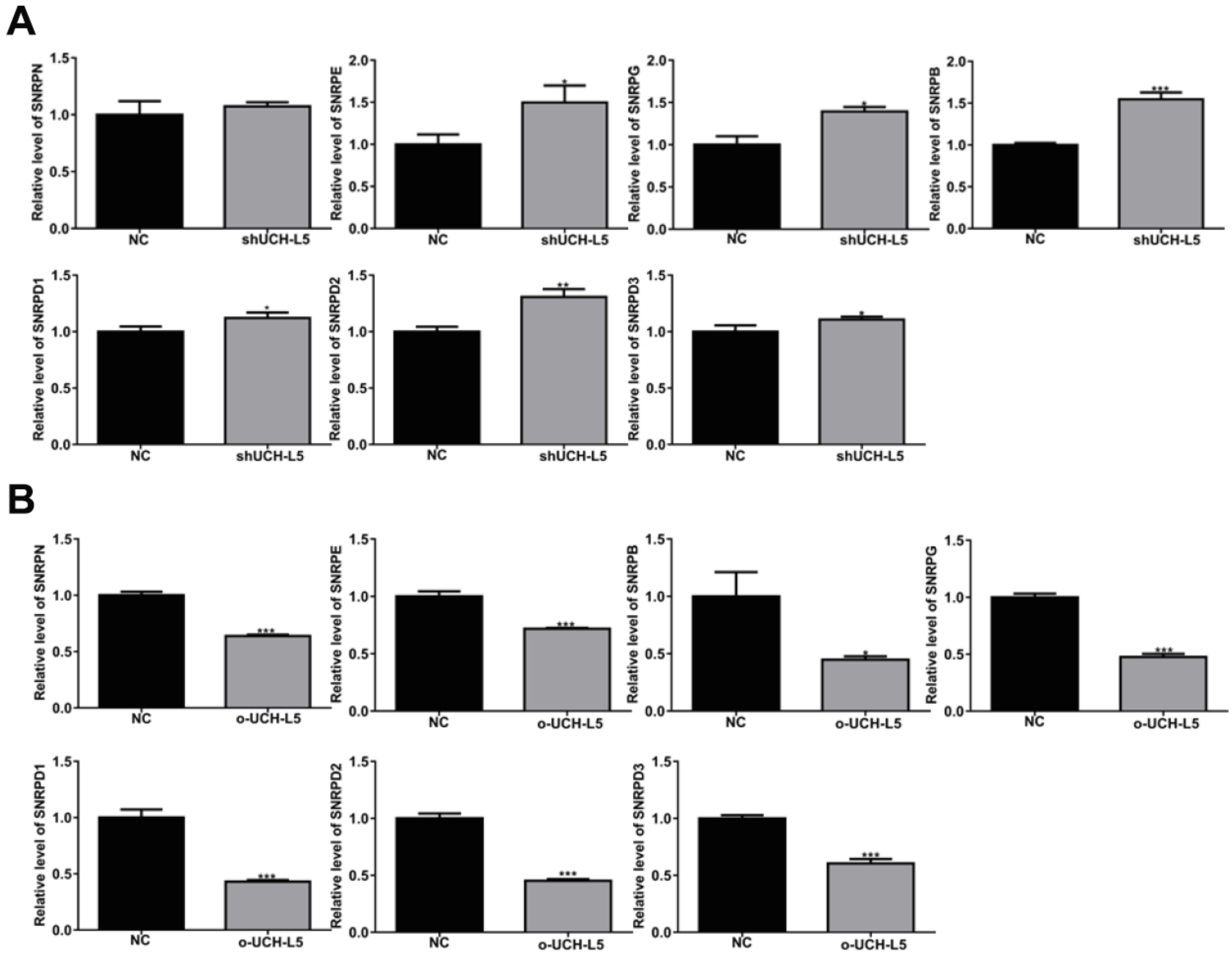

Supplementary Figure 1: UCH-L5 regulates mRNA expression of other Sm family members in U87MG cells with stable UCH-L5 silencing and UCH-L5 overexpressing by lentivirus. NC: negative control group, lentivirus stable infected but no knock-down or overexpression efficiency glioma cells. shUCH-L5: lentivirus stable knock-down UCH-L5 group. o-UCH-L5: Lentivirus stable overexpression UCH-L5 group. (**A**) mRNA expression of other Sm family members including SNRPN, SNRPB, SNRPE, SNRPG, SNRPD1 SNRPD2 SNRPD3 were detected in Lentivirus stable interfering UCH-L5 U87MG cells by RT qPCR, $^{**}P < 0.01$, $^{***}P < 0.001$. (**B**) mRNA expression of other Sm family members were detected in Lentivirus stable overexpression UCH-L5 U87MG cells by RT qPCR. $^{*}P < 0.05$, $^{**}P < 0.01$, $^{***}P < 0.001$.
